# Association of Baxter's Neuropathy and Fatty Infiltration of the Abductor Digiti Minimi Muscle on Magnetic Resonance Imaging: A Systematic Review

**DOI:** 10.1002/jfa2.70075

**Published:** 2025-08-20

**Authors:** John S. C. Chen, Mandy Abbott, Karl B. Landorf

**Affiliations:** ^1^ Discipline of Podiatry School of Allied Health Human Services and Sport La Trobe University Victoria Australia; ^2^ School of Health and Life Sciences Glasgow Caledonian University Glasgow UK

**Keywords:** abductor digiti minimi, Baxter's neuropathy, fatty atrophy, fatty infiltration, magnetic resonance imaging, plantar fasciitis, plantar heel pain

## Abstract

**Background:**

Fatty infiltration—or fatty atrophy—of the abductor digiti minimi (ADM) muscle of the foot on magnetic resonance imaging (MRI) has been attributed to entrapment of the first branch of the lateral plantar nerve (i.e., Baxter's neuropathy), a condition associated with plantar heel pain (PHP). The aim of this study was to investigate the evidence relating to the association between fatty infiltration of ADM and Baxter's neuropathy.

**Methods:**

This systematic review conducted searches in MEDLINE, CINAHL, SPORTDiscus, Embase and the Cochrane Library from 20^th^ June 2023 to 19^th^ of March 2024. Peer‐reviewed articles of retrospective, cross‐sectional observational, or cohort studies written in English that investigated the prevalence or frequency of fatty infiltration of ADM on MRI in adult participants were included. Study quality and risk of bias were assessed using the National Institutes of Health quality assessment tool for observational cohort and cross‐sectional studies.

**Results:**

Four studies (1052 participants) were identified and included in the review. Two studies were retrospective studies and two studies were cross‐sectional observational studies. Only one study was rated ‘good’ on quality assessment. The reported prevalence of fatty infiltration of ADM on MRI was reported to be between 4% and 11% in the general population. Prevalence was also reported to be similar in people with and without generalised foot pain (approximately 8% and 6%, respectively). No studies reported prevalence in specific populations with PHP or with Baxter's neuropathy.

**Conclusion:**

The association between fatty infiltration of ADM on MRI and entrapment of the first branch of the lateral plantar nerve as part of PHP still remains unknown due to the lack of robust evidence. Additional high‐quality studies investigating the association between PHP and fatty infiltration of ADM on MRI would be worthwhile to improve our understanding of the diagnostic value of MRI for this condition, which may aid decision‐making for the treatment of PHP, particularly surgical treatment of Baxter's neuropathy.

**Abbreviations**
ADM: abductor digiti minimi
BMI: body mass index
MRI: magnetic resonance imaging
NIH: National Institutes of Health
PHP: plantar heel pain
PRISMA: preferred reporting items for systematic reviews and meta‐analyses

## 1 Introduction

It is reported that up to 20% of plantar heel pain (PHP) may be of neural origin related to entrapment of the first branch of the lateral plantar nerve, which is commonly referred to as Baxter's neuropathy [1]. The first branch of the lateral plantar nerve is a mixed motor and sensory nerve that innervates abductor digiti minimi (ADM), flexor digitorum brevis and quadratus plantae, as well as providing sensory branches to the calcaneal periosteum and long plantar ligament [2, 3]. Currently, diagnosis of Baxter's neuropathy is not feasible using non‐invasive testing such as nerve conduction studies, so fatty infiltration (or fatty atrophy) of ADM on magnetic resonance imaging (MRI) has been hypothesised to be a surrogate marker of the condition [4].

The theory behind this fatty infiltration hypothesis is that neuropathy of the first branch of the lateral plantar nerve—due to conditions associated with PHP such as plantar fasciopathy or plantar heel spur—leads to atrophy of ADM muscle tissue, which over time is replaced with fat. Although, evidence supporting whether fatty infiltration of ADM is truly a sign of Baxter's neuropathy is lacking. Several case studies have reported fatty infiltration of ADM on MRI in individuals with plantar fasciitis [5, 6, 7, 8] and two additional case studies reported fatty infiltration occurring bilaterally [9, 10]. However, case studies provide very low‐level evidence as they do not use a control group to compare to, so they are at risk of confounding and bias. Consequently, their findings need to be viewed cautiously. Fatty infiltration of skeletal muscles has also been reported in individuals unrelated to nerve entrapment and especially in those with high body mass index (BMI) [11], diabetic peripheral neuropathy [12] and rheumatoid arthritis [13], so studies should also account for such factors, which case studies do not.

In addition to the very low‐level evidence currently available, there is no definitive diagnostic test for Baxter's neuropathy, so accurate diagnosis is difficult, and relies predominantly on the location and type of symptoms. For example, it has been suggested that symptoms that are ‘burning’ in nature may be related to neuropathy [14]. Nevertheless, early in the course of PHP, and because of the difficulty testing for Baxter's neuropathy, most patients are treated similarly, whether they may or may not have Baxter's neuropathy. However, if symptoms do not improve in the medium to long term (e.g., after 4–6 months) with conservative treatment, and if fatty infiltration of ADM is detected on MRI, decompressive surgery to release the first branch of the lateral plantar nerve has been recommended [15]. Because the evidence for this recommendation appears to be based on poor evidence, a systematic review investigating Baxter's neuropathy and its association with fatty infiltration (or fatty atrophy) of ADM on MRI is needed.

Therefore, the aim of this study was to systematically review and synthesise the evidence relating to the association between fatty infiltration of ADM and Baxter's neuropathy.

## 2 Methods

This systematic review has been reported in accordance with the Preferred Reporting Items for Systematic Reviews and Meta‐analyses (PRISMA) guidelines [16].

### 2.1 Search Strategy

Searches were conducted in MEDLINE, CINAHL, Scopus and SPORTDiscus from the 20th of June 2023 to the 19th of March 2024—Supporting Information S1. Google Scholar was used to track relevant citations and reference lists were screened to include additional studies not identified in the initial search.

### 2.2 Eligibility Criteria

Eligible studies had to be published in peer‐reviewed journals and written in the English Language. Studies had to be retrospective, cross‐sectional observational or cohort studies that examined the prevalence or frequency of fatty infiltration of ADM on MRI in a group of participants with foot pain (with or without an independent control group of participants without foot pain). Initial exploratory searches found no previous studies had recruited participants specifically with PHP, hence the search was widened to include individuals with more generalised foot pain instead. In addition, due to the paucity of research in this area, the eligibility criteria were widened to include all relevant studies for review; thus, limiters on the year of publication and restriction of studies to adult only participants were not imposed. Studies were excluded if they utilised medical imaging modalities other than MRI to examine the frequency of fatty infiltration of ADM. Case studies and case series articles were excluded as they do not have control groups and provide very low‐level evidence. Furthermore, review articles and literature reviews were excluded as only primary research was of interest.

### 2.3 Study Selection

Search results were exported from the bibliographic databases into Refworks (ProQuest Ex Libris, Malha Technology Park, Jerusalem) and duplicate citations were removed. All study titles and abstracts were examined by the first author (Chen) and studies deemed ineligible were excluded. The remaining full text articles were examined against eligibility criteria for inclusion in the literature review.

### 2.4 Data Extraction and Analysis

Extraction of individual participant (e.g., sex and age) and study characteristics (i.e., MRI field strength and protocol) were completed by the first author (Chen) and checked by the third author (Landorf). The primary themes included: methodological quality of studies, MRI protocols, and the frequency of fatty infiltration of ADM in participants with and without foot pain. Themes related to the methodology of studies such as blinding, were also extracted for quality appraisal. Because of the heterogeneity in methodology and outcome measures between studies, a meta‐analysis could not be performed.

### 2.5 Quality Appraisal

The National Institutes of Health (NIH) quality assessment tool for observational cohort and cross‐sectional studies was used to assess study quality and risk of bias [17]. Quality assessment of included studies was conducted by the first author (Chen) and checked by the third author (Landorf). The tool consists of 14 questions that are specific to observational and cross‐sectional studies, which encompass the key issues (‘concepts’) that determine internal validity of a study (selection, information, measurement and confounding bias). Once these issues have been assessed with the tool, it allows a quality rating to be applied to a study (rated as ‘poor’, ‘fair’ or ‘good’) based on individual details and consideration of the concepts, rather than a tally scoring system. *Low* risk of bias equates to a ‘good’ quality rating, whereas *high* risk of bias equates to a ‘poor’ quality rating.

## 3 Results

### 3.1 Study Characteristics

**FIGURE 1:**
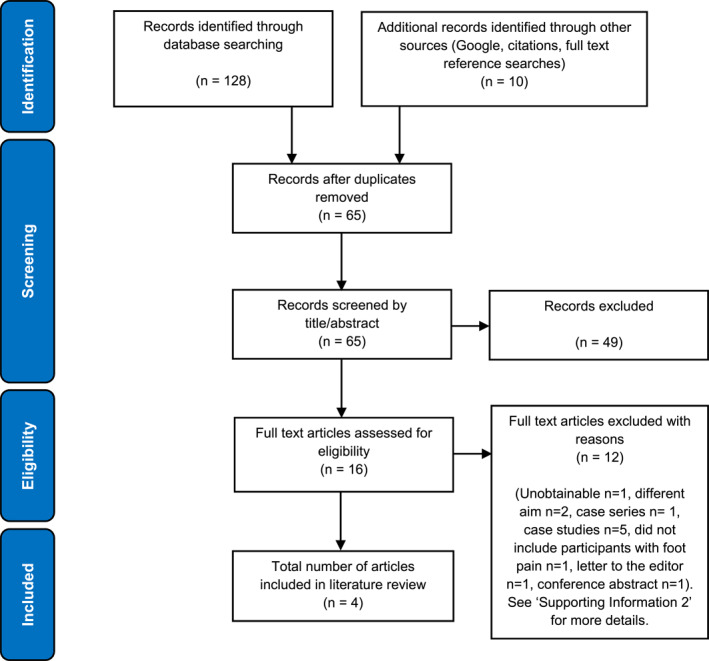
Preferred Reporting Items for Systematic reviews and Meta‐Analyses (PRISMA) flow diagram (adapted from Moher et al. (2009) The PRISMA group).

The database search identified a total of 65 unique citations of which 4 studies met inclusion criteria for review [18, 19, 20, 21]—Figure 1. The excluded studies and reasons for exclusion following full text article assessment are presented in Supporting Information S2.

For the 4 included studies there was a total sample size of 1052 participants; 972 participants with foot pain (66% female, mean age 48 years) and 80 control participants without foot pain (52.5% female, mean age 48 years)—Table 1.

**TABLE 1:** Study and participant characteristics.

Authors (date)	Study type	Population participants recruited from	Participant characteristics Mean age (range) Gender (%female/%male)	Sample size *n* = participants with foot pain	Assessor blinding
Chundru et al. (2008)	Retrospective	Individuals from the USA referred for MRI of the hindfoot due to presence of foot pain.	49.0 (10–92 years) (69.5/30.5)	200	Blinded
Recht et al. (2007)^a^	Cross‐sectional observational	NR (likely to be community‐dwelling USA).	NR (64.3/35.7)	602	Unblinded
Rodrigues et al. (2015)	Retrospective	NR (likely to be community‐dwelling Brazil).	49.2 (NR) (78.8/21.2)	90	Unblinded
Schmid et al. (2009)^b^	Cross‐sectional observational	NR (likely to be community‐dwelling Switzerland).	48.0 (20–86 years) (52.5/47.5)	80 (plus 80 controls)	Blinded

Abbreviation: NR, not reported.

^a^ Study did not report age and was not included in mean age calculations.

^b^ Only study that recruited an independent control group of participants without foot pain.

### 3.2 Quality Appraisal and Risk of Bias

Overall, there were several issues with the studies in relation to the quality assessment, which could have increased the risk of bias. Only two of the included studies utilised assessors who were blinded and three studies did not adequately report the population from which they recruited from. In addition, one study did not report the participant recruitment time period, three studies did not clearly report inclusion and exclusion criteria, and two studies did not report statistical analysis for adjustment of potential confounding variables. In summary, most studies were rated as having poor methodological quality [18, 19, 20], with only one study rated as ‘good quality’ [21]. Details of the quality appraisal for each study are included in Supporting Information S3.

### 3.3 Study Designs

All studies were either retrospective or cross‐sectional observational studies. Of these, two retrospectively reviewed MRI scans of participants with fatty infiltration of ADM on MRI to determine the prevalence of MRI findings that may be associated with Baxter's neuropathy (e.g., plantar fasciopathy, plantar calcaneal spur and hindfoot varicosities) [18, 20], one prospectively identified participants with fatty infiltration of ADM from consecutive hindfoot MRI examinations over a period of four months and reviewed patient notes for associated findings [19], and one identified the prevalence of fatty infiltration of ADM on MRI in participants with and without foot pain [21]. Two studies did not clearly describe the participant characteristics [18, 19] and three did not report the geographical location the study took place in [18, 19, 20], which means it is difficult to determine the populations from which the samples were recruited from.

Three studies reported the inclusion of participants with foot pain regardless of the underlying cause [18, 19, 21]. However, one study did not clearly specify the type of foot pain or state the impact of foot pain on participants [20], and therefore, it was not possible to determine the clinical significance of their findings in relation to entrapment of the first branch of the lateral plantar nerve. A single study reported only one participant had a clinical diagnosis of possible nerve entrapment prior to their MRI scan [19], but besides this, none of the studies focused on the recruitment of participants with PHP. A single study recruited an independent control group without foot pain, and matched the no foot pain group to the foot pain group for age and sex [21]. This study described their study population but failed to exclude participants with endocrine or neurological conditions, which may have confounded their results due to, for example, peripheral neuropathy. BMI was not reported for all studies, and for three studies [18, 19, 20], it was unclear whether participants had underlying comorbidities such as inflammatory arthropathies, endocrine or neurological conditions, previous surgery or trauma that affected their lower limb sensation or ability to walk and run.

### 3.4 MRI Protocol

All studies utilised MRI to assess fatty infiltration of ADM in participants with foot pain, one of which also assessed participants without foot pain [21]. One study clearly described their MRI protocol and equipment specifications [21], but the remaining three studies [18, 19, 20] lacked MRI protocol descriptions with no reporting of the manufacturer of the MRI unit, coil type or sequence specifications, which leads to reproducibility and generalisability issues—Table 2.

**TABLE 2:** MRI protocol and equipment specification.

Authors (date)	Field strength	Manufacturer	Coil	Sequences	Interpreter
Chundru et al. (2008)	0.2–1.5T	NR	NR	T1‐weighted, sagittal short‐tau inversion (STIR), oblique axial STIR, coronal T1‐weighted, coronal STIR, plantar flexed axial proton density T2‐weighted axial (with fat saturation or STIR)	2 radiologists (each with 4.5 years experience)
Recht et al. (2007)	0.2–1.5T	NR	NR	T1‐weighted axial, coronal T2‐weighted axial, coronal and sagittal (with or without fat suppression) and STIR	1 of 2 radiologists (fellowship trained)
Rodrigues et al. (2015)	1.5T	NR	NR	T1‐weighted sagittal sequences and PDW with fat suppression T2‐weighted axial, coronal/oblique PDW with fat suppression	NR
Schmid et al. (2009)	1.5T	Siemens Medical Solutions	Circularly polarised send‐receive extremity coil	T1‐weighted coronal images T2‐weighted coronal images	2 radiologists (with 5 and 20 years of experience)

Abbreviations: NR, not reported; T, Tesla.

Two studies used 1.5 Tesla (T) magnetic field strength scans [20, 21] and another two studies adopted a variety of magnet strengths ranging from 0.2 to 1.5 T [18, 19]. All studies reported using T1‐weighted and T2‐weighted sequences, but their sequence specifications were not described in detail in three out of the four studies [18, 19, 20]. In general, there was a lack of consistency in MRI protocol reporting across all the studies. Regarding assessment of the MR images, three studies recruited musculoskeletal radiologists with experience ranging from 4.5 to 20 years to interpret MRI scans for fatty infiltration of ADM prevalence [18, 19, 21]. However, one study did not report assessor details [20] leading to uncertainty in the validity of the findings reported. Two studies did not report whether assessors were blinded [19, 20].

### 3.5 Frequency and Extent of Fatty Infiltration of ADM

The prevalence of fatty infiltration of ADM using MRI in participants with foot pain was reported in three studies [18, 19, 21], and of these, two measured the extent of fatty infiltration of ADM using graded classification scales [19, 21]. Another study did not report prevalence of fatty infiltration of ADM [20], but instead, focused on associations of fatty infiltration of ADM with other pathological findings on MRI (i.e., plantar fasciitis, calcaneal spur, etc.) related to Baxter's neuropathy. Furthermore, this study purposively sampled participants with grade IV fatty atrophy of ADM (i.e., more fat than muscle present), based on the Goutallier and Bernageau classification [22]. A comparison of grading scales used in each study is presented in Table 3.

**TABLE 3:** Comparison of grading scales for fatty infiltration of ADM on MRI between included studies.

Authors (date)	Grade 0	Grade 1	Grade 2	Grade 3	Grade 4
Chundru et al. (2008)^a^					
Schmid et al. (2009)	Normal muscle	Mild fatty atrophy with more muscle than fat	Substantial fatty atrophy with more fat than muscle or equal parts of fat and muscle	No grade 3 in this scale	No grade 4 in this scale
Recht et al. (2007)	No fat or minimal fatty streaks	Increased fat within the muscle but greater amount of muscle	Equal amounts of fat and muscle	Greater amount of fat than muscle	No grade 4 in this scale
Rodrigues et al. (2015)^b^	Completely normal without any fatty streaks	Muscle contains some fatty streaks	Fatty infiltration is important, but there is more muscle than fat	Equal amounts of fat and muscle	More fat than muscle is present

^a^ Study did not use grading scale (i.e., they only assessed for presence or absence of fatty infiltration).

^b^ Study used Goutallier et al. (Goutallier et al., 1994) grading scale to grade fatty infiltration of ADM on MRI.

One study reported a prevalence of 5.6% (100/1780) for fatty infiltration of ADM after reviewing 1780 consecutive MRI scans of participants with foot pain [18]. Another study prospectively recruited a large sample of 602 consecutive participants with foot pain and found a prevalence of 6.3% (38/602) for fatty infiltration of ADM on MRI [19]. In this study, 21% (8/38) had grade 1 atrophy, 29% (11/38) had grade 2 atrophy, and 50% (19/38) had grade 3 atrophy. Only one study assessed prevalence of fatty infiltration of ADM in participants with and without foot pain and reported a similar prevalence of substantial (grade 2) fatty atrophy between the two groups (approximately 8% and 6%, respectively) [21]—Table 4.

**TABLE 4:** Summary of findings for prevalence on MRI of fatty infiltration of ADM in participants with and without foot pain.

Authors (date)	Population (sample size)	MRI findings
Chundru et al. (2008)	Foot pain (*n* = 200)	Prevalence of fatty atrophy of ADM found to be 5.6% (100/1780)^a^
Recht et al. (2007)	Foot pain (*n* = 602)	Fatty atrophy of ADM is not a rare finding with a reported prevalence of 6.3%. Its clinical relevance as seen on MRI is uncertain.
Rodrigues et al. (2015)	Foot pain (*n* = 90)	This study did not report prevalence of fatty atrophy of ADM.
Schmid et al. (2009)	Foot pain (*n* = 80) Control (*n* = 80)	Prevalence of substantial (grade 2) fatty atrophy of the ADM muscle was found to be similar in both patients with foot pain (8%) and those without foot pain (6%).

^a^ This study reviewed 1780 MRI studies in order to identify 100 individuals with fatty infiltration (or atrophy) of ADM, hence the prevalence calculation. An additional 100 individuals were recruited without fatty infiltration of ADM on MRI. So overall, 200 individuals with foot pain were included in the study with and without fatty infiltration of ADM.

## 4 Discussion

This systematic review aimed to determine what is currently known about fatty infiltration of ADM on MRI in cases where Baxter's neuropathy may be present. It included two retrospective and two cross‐sectional observational studies and is the first systematic review on this topic. This review found that the association between fatty infiltration of ADM on MRI and entrapment of the first branch of the lateral plantar nerve as part of PHP still remains unknown due to a lack of robust evidence. This finding is in contrast to what has been suggested by several authors who have detected this finding on MRI. For example, Dirim and colleagues published their observations of fatty infiltration of ADM on MRI in a case study of one 42 year old woman with bilateral PHP [9]. They attributed their finding to Baxter's neuropathy, where the first branch of the lateral plantar nerve becomes compressed. They went on to hypothesise that entrapment of the first branch of the lateral plantar nerve can lead to fatty infiltration (or fatty atrophy) of the ADM muscle being visible on MRI [9]—a surrogate marker of entrapment of this nerve. This hypothesis has since been repeated by other authors, all of whom have published case studies [5, 6, 7, 8, 10]. However, case studies provide very low‐level evidence as they do not use a control group, so their findings need to be viewed cautiously as there may be confounding and bias present.

The first cross‐sectional study that recorded fatty infiltration of ADM on MRI in a large group of individuals with ‘heel pain syndrome’ was by Stanzcak and colleagues [4] who published their findings in 2001 in a conference abstract only (i.e., this study does not appear to have progressed to a full peer‐reviewed journal article). Unfortunately, because of the limited access to their detailed methods, their findings cannot be confidently discussed in this review. Since then, four additional studies (two retrospective and two cross‐sectional observational studies) have investigated fatty infiltration of ADM in individuals with generalised foot pain [18, 19, 20, 21]. The earliest of these, a cross‐sectional observational study published in 2007 by Recht and colleagues [19] recruited a large sample of 602 consecutive participants with foot pain from a variety of conditions (not just PHP) and reported a 6.3% (38/602) prevalence of fatty infiltration of ADM on MRI. The authors found that none of the participants with fatty infiltration of ADM presented with a possible diagnosis of Baxter's neuropathy (i.e., participants with fatty infiltration of ADM had no clinical signs and symptoms of entrapment of the first branch of the lateral plantar nerve). As a result, they concluded that fatty infiltration of ADM may be an incidental finding on MRI and unrelated to nerve entrapment.

A second cross‐sectional observational study was published in 2009 by Schmid and colleagues [21], which included a control group. They recruited 160 participants, including 80 with foot pain from a variety of conditions and 80 without foot pain who were matched for age and sex. They observed a similar prevalence of substantial fatty infiltration of ADM on MRI between those with and without foot pain (approximately 8% and 6%, respectively). As such, they found no significant differences in the degree of fatty infiltration of ADM on MRI between people with and without foot pain. However, Schmid and colleagues [21] did not account for increased BMI or abdominal obesity, so confounding due to these variables cannot be ruled out. Indeed, there is evidence to suggest that BMI can influence fatty infiltration of skeletal muscle [11]. Furthermore, none of the studies above checked to ensure the groups were similar for other important co‐morbidities that could have confounded their findings (e.g., medical conditions that may be associated with fat deposition).

Further to the two cross‐sectional studies discussed above, two retrospective studies have also investigated fatty infiltration of ADM in adults with foot pain from a variety of conditions [18, 20]. However, similar to the two cross‐sectional studies, these studies did not focus on individuals with PHP or Baxter's neuropathy specifically. The first of these studies published in 2008 by Chundru and colleagues [18] retrospectively reviewed 1780 MRI reports of patients with foot pain from a variety of conditions. They reported a similar prevalence in their sample to Recht et al. [19] of 5.6% for fatty infiltration of ADM. Following the calculation of prevalence from the 1780 MRI reports, they went on to further analyse 100 individuals with and 100 individuals without fatty infiltration of ADM. They found a significant association between fatty infiltration of ADM and advancing age, plantar calcaneal spur formation and plantar fasciopathy [18]. The second study published in 2015 by Rodrigues and colleagues [20] retrospectively reviewed MRI scans of 90 patients with grade IV atrophy (i.e., fatty infiltration) of ADM according to the grading scale proposed by Goutallier et al. [22] and observed fatty infiltration of ADM was most prevalent in females with foot pain. Furthermore, the authors found that there was a strong association between grade IV atrophy of ADM and ‘plantar fasciitis’ (i.e., plantar fasciopathy), as well as hindfoot varicosities [20]. However, both the study by Rodrigues and colleagues [20] and the one by Chundru and colleagues [18] did not use a control group (i.e., people without PHP/foot pain), so their findings may have been confounded by important variables that could have influenced the atrophy of ADM such as age, sex and other comorbidities.

With the information outlined above in mind, most research in this area consists of studies with poor methodological quality, which were at risk of confounding and bias. Moreover, no studies to date have focused on fatty infiltration of ADM on MRI as it relates to PHP or Baxter's neuropathy specifically (although, as stated previously Baxter's neuropathy is difficult to definitively diagnose). Previous cross‐sectional studies—as discussed above—have reported the prevalence of fatty infiltration of ADM on MRI in people with and without foot pain from a variety of conditions, but their findings cannot be confidently extrapolated to PHP and specifically, Baxter's neuropathy. This is a key finding of this review, and clinicians need to be highly cautious when interpreting the presence of fatty infiltration of ADM on MRI as a surrogate marker for Baxter's neuropathy when managing individuals with PHP, particularly if invasive interventions are being considered such as surgery. Future cross‐sectional studies, therefore, need to not only focus on PHP (rather than foot pain from a variety of conditions) but they need to include a comparison group without PHP that controls for variables such as age, sex and BMI/obesity to minimise confounding. In addition, studies higher in the evidence hierarchy, such as well‐controlled cross‐sectional observational or prospective cohort studies, would provide more certainty in relation to causality; that is, whether Baxter's neuropathy associated with PHP causes fatty infiltration of ADM. Finally, previous studies have used relatively low MRI field‐strengths of 0.2–1.5 T to generate images. In contrast, future studies ideally should use a 3.0 T (or greater) MRI scanner, which has a higher field‐strength and will result in better image quality for interpretation.

### 4.1 Limitations

This systematic review was designed to be a comprehensive review of the literature, however its findings should be considered with five limitations in mind. Firstly, and as highlighted above, previous studies recruited samples of participants with foot pain regardless of the underlying cause (i.e., not specifically PHP). Accordingly, these findings cannot be generalised to PHP generally or Baxter's neuropathy specifically. Secondly, only one author culled studies from the original search, and other data collection and quality assessment was not done independently with two assessors (it was done by one assessor with a second checking). Thirdly, there was substantial heterogeneity amongst all included studies and only one study was rated ‘good’ on quality assessment. Most studies were rated as having poor methodological quality and were poorly controlled for confounding variables. Only two of the included studies utilised assessors who were blinded, which could lead to assessment bias and consequently, the results from these studies need to be considered cautiously. Fourthly, BMI and abdominal obesity were not reported in all studies, which could have confounded fatty infiltration of ADM as it relates to foot pain (i.e., the fatty infiltration of ADM reported may have simply been due to obesity). Lastly, the majority of studies did not report intra‐ and inter‐assessor reliability for grading fatty infiltration of ADM on MRI, which may have affected the accuracy of the imaging observations. Ideally, assessors should demonstrate both intra‐ and inter‐assessor reliability, which is something future studies should determine prior to data collection.

## 5 Conclusions

This systematic review investigated what is currently known about the association between fatty infiltration of ADM on MRI and Baxter's neuropathy. The association between fatty infiltration of ADM on MRI and entrapment of the first branch of the lateral plantar nerve as part of PHP still remains unknown due to the lack of robust evidence. Thus, clinicians who are involved in the assessment and management of PHP, should be highly cautious when interpreting the presence of fatty infiltration of ADM on MRI as a surrogate marker for Baxter's neuropathy because it may be an incidental finding unrelated to nerve entrapment. Additional high‐quality studies investigating the frequency of fatty infiltration of the ADM on MRI in people with and without PHP (rather than generalised foot pain), whilst controlling for important confounding variables such as obesity, are needed.

## Author Contributions

**John S. C. Chen:** investigation, methodology, formal analysis, writing – original draft preparation, writing – review and editing. **Mandy Abbott**: supervision, writing – review and editing. **Karl B. Landorf:** supervision, validation, formal analysis, writing – review and editing.

## Ethics Statement

The authors have nothing to report.

## Consent

The authors have nothing to report.

## Conflicts of Interest

Karl B. Landorf is an Emeritus Editor and is a member of the Editorial Board of the Journal of Foot and Ankle Research. It is the journal policy that editors are removed from the peer review and editorial decision‐making processes for manuscripts they have co‐authored. Otherwise, the authors declare that they have no conflicts of interest.

## Supporting information


Supporting Information S1

Supporting Information S2

Supporting Information S3

## Data Availability

The datasets generated during and/or analysed during the current study are available from the corresponding author on reasonable request.
