# Supplementary material for: Association of Baxter's Neuropathy and Fatty Infiltration of the Abductor Digiti Minimi Muscle on Magnetic Resonance Imaging: A Systematic Review
Source: J Foot Ankle Res. 2025 Aug 20;18(3):e70075. doi: 10.1002/jfa2.70075 (PMC12367558; doi:10.1002/jfa2.70075)
Supplement: Supplementary file 1 — Supporting Information S1 [file JFA2-18-e70075-s001.docx]

**Supporting Information 1.** Database search terms

CINAHL (22)

1. plantar OR heel OR calcane* OR fasci* AND pain*
2. “baxter* neuro*” OR “baxter* nerv*”
3. “fat* atroph*” OR “fat* infiltrati*”
4. “magnetic resonance imaging” OR MR OR MRI
5. (1 OR 2)
6. 5 AND 3 AND 4

Medline (48)

1. plantar OR heel OR calcane* OR fasci* AND pain*
2. “baxter* neuro*” OR “baxter* nerv*”
3. “fat* atroph*” OR “fat* infiltrati*”
4. “magnetic resonance imaging” OR MR OR MRI
5. (1 OR 2)
6. 5 AND 3 AND 4

Scopus (44)

1. (plantar OR heel OR calcane* OR fasci* AND pain*) ti, ab, keywords
2. (“baxter* neuro*” OR “baxter* nerv*”) ti, ab, keywords
3. (“fat* atroph*” OR “fat* infiltrati*”) ti, ab, keywords
4. (“magnetic resonance imaging” OR MR OR MRI) ti, ab, keywords
5. (1 OR 2)
6. 5 AND 3 AND 4

Sportsdiscus (14)

1. plantar OR heel OR calcane* OR fasci* AND pain*
2. “baxter* neuro*” OR “baxter* nerv*”
3. “fat* atroph*” OR “fat* infiltrati*”
4. “magnetic resonance imaging” OR MR OR MRI
5. (1 OR 2)
6. 5 AND 3 AND 4
