# Supplementary material for: Association of Baxter's Neuropathy and Fatty Infiltration of the Abductor Digiti Minimi Muscle on Magnetic Resonance Imaging: A Systematic Review
Source: J Foot Ankle Res. 2025 Aug 20;18(3):e70075. doi: 10.1002/jfa2.70075 (PMC12367558; doi:10.1002/jfa2.70075)
Supplement: Supplementary file 2 — Supporting Information S2 [file JFA2-18-e70075-s002.docx]

**Supporting Information 2.** List of excluded articles and reasons

| **Authors (date)** | **Grounds for exclusion** |
| --- | --- |
| Andrade Fernandes de Mello et al (2017) | Did not include participants with foot pain. |
| Balius et al (2021) | Case series. Different aim and objectives. |
| Bas et al (2020) | Different aim and objectives. |
| Chimutengwende-Gordon et al (2014) | Case report. |
| Dirim et al (2010) | Case report. |
| Fleckenstein and Shellock (1991) | Review article. |
| Jaring et al (2019) | Case report. |
| Kaur et al (2024) | Case report. |
| Moreno Garcia et al (2017) | Letter to the editor about a case report. |
| Ong and Chin (2020) | Case report. |
| Rajmane et al (2003) | Unobtainable. Conference presentation. |
| Stanczak et al (2001) | Unobtainable – only a conference abstract (no full article published). |
