# Supplementary material for: Association of Baxter's Neuropathy and Fatty Infiltration of the Abductor Digiti Minimi Muscle on Magnetic Resonance Imaging: A Systematic Review
Source: J Foot Ankle Res. 2025 Aug 20;18(3):e70075. doi: 10.1002/jfa2.70075 (PMC12367558; doi:10.1002/jfa2.70075)
Supplement: Supplementary file 3 — Supporting Information S3 [file JFA2-18-e70075-s003.docx]

**Supporting Information 3.** NIH Quality Assessment Tool for observational cohort and cross-sectional studies.

| Study: **Chundru et al (2008)** |
| --- |

| Criteria | Yes | No | Other (CD, NR, NA)* |
| --- | --- | --- | --- |
| 1. Was the research question or objective in this paper clearly stated? | * |  |  |
| 2. Was the study population clearly specified and defined? | * |  |  |
| 3. Was the participation rate of eligible persons at least 50%? |  |  | NR |
| 4. Were all the subjects selected or recruited from the same or similar populations (including the same time period)? Were inclusion and exclusion criteria for being in the study prespecified and applied uniformly to all participants? |  | * | Limited mention of inclusion and exclusion criteria. Source of participants unclear. |
| 5. Was a sample size justification, power description, or variance and effect estimates provided? |  | * |  |
| 6. For the analyses in this paper, were the exposure(s) of interest measured prior to the outcome(s) being measured? |  | * | Retrospective study |
| 7. Was the timeframe sufficient so that one could reasonably expect to see an association between exposure and outcome if it existed? |  | * | Retrospective study |
| 8. For exposures that can vary in amount or level, did the study examine different levels of the exposure as related to the outcome (e.g., categories of exposure, or exposure measured as continuous variable)? |  | * |  |
| 9. Were the exposure measures (independent variables) clearly defined, valid, reliable, and implemented consistently across all study participants? | * |  |  |
| 10. Was the exposure(s) assessed more than once over time? |  | * |  |
| 11. Were the outcome measures (dependent variables) clearly defined, valid, reliable, and implemented consistently across all study participants? | * |  |  |
| 12. Were the outcome assessors blinded to the exposure status of participants? | * |  |  |
| 13. Was loss to follow-up after baseline 20% or less? |  | * | Retrospective study |
| 14. Were key potential confounding variables measured and adjusted statistically for their impact on the relationship between exposure(s) and outcome(s) |  | * |  |

| **Quality Rating (Good, Fair, or Poor)** |
| --- |
| Poor |
| Additional Comments (If POOR, please state why):  Source of participants unclear, inclusion/exclusion criteria unclear, no adjustment for confounding variables. |

*CD, cannot determine; NA, not applicable; NR, not reported

| Study: **Recht et al (2007)** |
| --- |

| Criteria | Yes | No | Other (CD, NR, NA)* |
| --- | --- | --- | --- |
| 1. Was the research question or objective in this paper clearly stated? | * |  |  |
| 2. Was the study population clearly specified and defined? |  | * |  |
| 3. Was the participation rate of eligible persons at least 50%? |  |  | NR |
| 4. Were all the subjects selected or recruited from the same or similar populations (including the same time period)? Were inclusion and exclusion criteria for being in the study prespecified and applied uniformly to all participants? |  | * | Inclusion/exclusion criteria unclear  Source of participants unclear |
| 5. Was a sample size justification, power description, or variance and effect estimates provided? |  | * |  |
| 6. For the analyses in this paper, were the exposure(s) of interest measured prior to the outcome(s) being measured? |  | * | Cross-sectional study |
| 7. Was the timeframe sufficient so that one could reasonably expect to see an association between exposure and outcome if it existed? |  | * | Cross-sectional study |
| 8. For exposures that can vary in amount or level, did the study examine different levels of the exposure as related to the outcome (e.g., categories of exposure, or exposure measured as continuous variable)? |  | * |  |
| 9. Were the exposure measures (independent variables) clearly defined, valid, reliable, and implemented consistently across all study participants? | * |  |  |
| 10. Was the exposure(s) assessed more than once over time? |  | * | Cross-sectional study |
| 11. Were the outcome measures (dependent variables) clearly defined, valid, reliable, and implemented consistently across all study participants? | * |  |  |
| 12. Were the outcome assessors blinded to the exposure status of participants? |  |  | NA |
| 13. Was loss to follow-up after baseline 20% or less? |  | * | Cross-sectional study |
| 14. Were key potential confounding variables measured and adjusted statistically for their impact on the relationship between exposure(s) and outcome(s) |  | * | NR – no mention of statistical analysis |

| **Quality Rating (Good, Fair, or Poor)** |
| --- |
| Poor |
| Additional Comments (If POOR, please state why):  Source and selection of participants unclear, inclusion/exclusion criteria unclear, no blinding of assessors, no information regarding statistical analysis in methods or results, no adjustment for confounding variables. |

*CD, cannot determine; NA, not applicable; NR, not reported

| Study: **Rodrigues et al (2015)** |
| --- |

| Criteria | Yes | No | Other (CD, NR, NA)* |
| --- | --- | --- | --- |
| 1. Was the research question or objective in this paper clearly stated? | * |  |  |
| 2. Was the study population clearly specified and defined? |  | * |  |
| 3. Was the participation rate of eligible persons at least 50%? |  |  | NR |
| 4. Were all the subjects selected or recruited from the same or similar populations (including the same time period)? Were inclusion and exclusion criteria for being in the study prespecified and applied uniformly to all participants? |  | * | Source and selection of participants unclear  No recruitment time period mentioned  Inclusion/exclusion criteria unclear |
| 5. Was a sample size justification, power description, or variance and effect estimates provided? |  | * |  |
| 6. For the analyses in this paper, were the exposure(s) of interest measured prior to the outcome(s) being measured? |  | * | Retrospective study |
| 7. Was the timeframe sufficient so that one could reasonably expect to see an association between exposure and outcome if it existed? |  | * | Retrospective study |
| 8. For exposures that can vary in amount or level, did the study examine different levels of the exposure as related to the outcome (e.g., categories of exposure, or exposure measured as continuous variable)? |  | * |  |
| 9. Were the exposure measures (independent variables) clearly defined, valid, reliable, and implemented consistently across all study participants? | * |  |  |
| 10. Was the exposure(s) assessed more than once over time? |  | * | Retrospective study |
| 11. Were the outcome measures (dependent variables) clearly defined, valid, reliable, and implemented consistently across all study participants? | * |  |  |
| 12. Were the outcome assessors blinded to the exposure status of participants? |  |  | NA |
| 13. Was loss to follow-up after baseline 20% or less? |  | * | Retrospective study |
| 14. Were key potential confounding variables measured and adjusted statistically for their impact on the relationship between exposure(s) and outcome(s) |  | * |  |

| **Quality Rating (Good, Fair, or Poor)** |
| --- |
| Poor |
| Additional Comments (If POOR, please state why):  Source and selection of participants unclear, no recruitment time period, inclusion/exclusion criteria unclear, no blinding of assessors, no adjustment for confounding variables. |

*CD, cannot determine; NA, not applicable; NR, not reported

| Study: **Schmid et al (2009)** |
| --- |

| Criteria | Yes | No | Other (CD, NR, NA)* |
| --- | --- | --- | --- |
| 1. Was the research question or objective in this paper clearly stated? | * |  |  |
| 2. Was the study population clearly specified and defined? | * |  |  |
| 3. Was the participation rate of eligible persons at least 50%? |  |  | NR |
| 4. Were all the subjects selected or recruited from the same or similar populations (including the same time period)? Were inclusion and exclusion criteria for being in the study prespecified and applied uniformly to all participants? | * |  |  |
| 5. Was a sample size justification, power description, or variance and effect estimates provided? |  | * |  |
| 6. For the analyses in this paper, were the exposure(s) of interest measured prior to the outcome(s) being measured? |  | * | Cross-sectional study |
| 7. Was the timeframe sufficient so that one could reasonably expect to see an association between exposure and outcome if it existed? |  | * | Cross-sectional study |
| 8. For exposures that can vary in amount or level, did the study examine different levels of the exposure as related to the outcome (e.g., categories of exposure, or exposure measured as continuous variable)? |  | * |  |
| 9. Were the exposure measures (independent variables) clearly defined, valid, reliable, and implemented consistently across all study participants? | * |  |  |
| 10. Was the exposure(s) assessed more than once over time? |  | * | Cross-sectional study |
| 11. Were the outcome measures (dependent variables) clearly defined, valid, reliable, and implemented consistently across all study participants? | * |  |  |
| 12. Were the outcome assessors blinded to the exposure status of participants? | * |  |  |
| 13. Was loss to follow-up after baseline 20% or less? |  | * | Cross-sectional study |
| 14. Were key potential confounding variables measured and adjusted statistically for their impact on the relationship between exposure(s) and outcome(s) | * |  | Matched controls (age, gender) |

| **Quality Rating (Good, Fair, or Poor)** |
| --- |
| Good |
| Additional Comments (If POOR, please state why):  Did not control for BMI and also did not specifically recruit participants with PHP. |

*CD, cannot determine; NA, not applicable; NR, not reported
